# Leading Pathogens Involved in Co-Infection and Super-Infection with COVID-19: Forensic Medicine Considerations after a Systematic Review and Meta-Analysis

**DOI:** 10.3390/pathogens12050646

**Published:** 2023-04-27

**Authors:** Roberto Scendoni, Emanuele Bury, Isabella Lima Arrais Ribeiro, Mariano Cingolani, Roberto Cameriere, Anna De Benedictis, Francesco De Micco

**Affiliations:** 1 Department of Law, University of Macerata, 62100 Macerata, Italy; 2 Postgraduate Program in Dentistry, Federal University of Paraíba, Campus I, João Pessoa 58051-900, Brazil; 3 Department of Medicine and Health Sciences, University of Molise, 86100 Campobasso, Italy; 4 Fondazione Policlinico Universitario Campus Bio-Medico, 00128 Roma, Italy; 5 Research Unit of Nursing Science, Department of Medicine and Surgery, Università Campus Bio-Medico di Roma, 00128 Roma, Italy; 6 Research Unit of Bioethics and Humanities, Department of Medicine and Surgery, Università Campus Bio-Medico di Roma, 00128 Roma, Italy

**Keywords:** COVID-19, co-infection, super-infection, forensic pathology, forensic medicine, autopsy findings

## Abstract

The COVID-19 pandemic raised concerns about the potential for co-infection or over-infection with other respiratory infections, as they can complicate the diagnosis, treatment and prognosis of the disease. This is also a challenge for forensic pathologists, who may come across cases where the presence of co-infection or over-infection is suspected or confirmed, and it is important that they take this into account when determining the cause of death. The aim of this systematic review is to analyse the prevalence of each specific pathogen co-infecting or over-infecting patients with SARS-CoV-2 infection. In total, 575 studies were selected from the Scopus and Pub-Med online databases and 8 studies were included in a meta-analysis. Male gender, advanced age and nursing home care are risk factors associated with the development of co-infection, whereas age, tachypnoea, hypoxaemia and bacterial infection are predictors of mortality. Overall, however, having a SARS-CoV-2 infection does not represent a real risk for the development of co-infections/super-infections.

## 1. Introduction

From 2020 to date, the COVID-19 pandemic has had a significant impact on health systems worldwide. The respiratory disease brought about by SARS-CoV-2 infection can manifest itself with varying severity: symptomatic or paucisymptomatic infection, severe bilateral pneumonia up to acute respiratory distress (ARDS) with diffuse alveolar damage (DAD) [1]. Non-invasive or invasive mechanical ventilation and intensive care unit (ICU) admission are necessary for the treatment of the most severe forms of the disease [2]. Nevertheless, serious complications related to extensive lung damage, extra-pulmonary disease or the use of mechanical support systems can occur. [3]. In this scenario, COVID-19 can be complicated by bacterial and fungal co-infections and super-infections resulting in increased morbidity and mortality, particularly in critically ill patients [4].

Co-infection is a simultaneous infection of a host by more than one pathogen species; it occurs ≤48–72 h after admission in 7% of patients and is uncommon in COVID-19 patients [5]. A super-infection is a secondary infection that overlaps with a previous infection 48–72 h after admission and is most frequently diagnosed in critically ill patients [6]. In both cases, microbial pathogenesis could be augmented, resulting in increased morbidity and mortality of viral infections [7].

Bacterial co-infection and super-infection concerns in COVID-19 patients have led to the widespread use of antibiotics empirically in both hospital and community settings [8]. The significant increase in antibiotic prescriptions during the pandemic challenges antimicrobial stewardship programs and risks the emergence of multi-drug resistant bacteria, with their associated impact on morbidity, mortality and costs [9].

The most common co-pathogens identified were Klebsiella pneumoniae, Pseudomonas aeruginosa, Haemophilus influenzae, Escherichia coli, Acinetobacter baumannii, Mycoplasma pneumoniae and Staphylococcus aureus. [4,10]. The most frequent pathogens responsible for fungal co-infections and super-infections were *Aspergillus* spp., *Candida* spp., *Mucorales*, *Histoplasma* spp., *Cryptococcus* spp. and Pneumocystis jirovecii. [11].

The combined approach of clinic, laboratory tests and imaging makes it possible to differentiate severe viral pneumonia from bacterial and fungal co-infection/super-infection [12].

Bacterial co-infections in COVID-19 are a widespread concern among healthcare professionals [13]. Co-infections and super-infections can exacerbate the severity of COVID-19 symptoms, impact the efficacy of treatments, increase the risk of long-term complications and raise the risk of mortality [14]. Thus, in addition to a clinical perspective, the topic is of special interest for forensic pathology. Forensic pathologists should be aware of the possibilities of co-infections and super-infections and consider these factors when analysing autopsy findings and determining the cause of death. In addition, there may be cases in which a patient with a pre-existing medical condition or an immunodeficiency contracted both COVID-19 and another infection; this may further complicate the interpretation of autopsy findings and require a more detailed examination. Post mortem examination plays an important role in identifying the causes of death, analysing complications, identifying patterns of lung injury, and evaluating the spread of the virus in the population in order to develop better therapeutic strategies and reduce the spread of the virus [15]. Establishing the cause of death of a patient with COVID-19 also provides relevant information for clinical risk management by identifying possible errors such as misdiagnosis, inappropriate treatment or avoidable complications with significant consequences for patient safety [16,17,18].

It is obvious that this field of research should be overseen through a strongly multidisciplinary approach in order to ensure a complete and accurate assessment. Co-working of experts from different disciplines can help identify relevant information and ensure the quality and impartiality of medical and legal assessments consistent with job well done ethics model [19]. One example is the findings concerning the way in which SARS-CoV-2 replicates within our cells, which also suggest a new potential drug target for new anti-COVID-19 drugs but, at the same time, resistant viruses emerge relatively easily [20]. However, membrane rearrangements related to the replication/transcription compartment of coronaviruses, in addition to being a target for potential antivirals, allow resistant viruses to emerge with relative ease [21,22].

The aim of this systematic review is to analyse the prevalence of each specific pathogen co-infecting or superinfecting patients with SARS-CoV-2 infection in order to offer forensic pathologists, but not only, an up-to-date tool for determining the cause of death in COVID-19 patients.

## 2. Materials and Methods

### 2.1. Study Design

This systematic review and meta-analysis followed the Preferred Reporting Items for Systematic Reviews and Meta-analyses (PRISMA) reporting guideline [23]. The study protocol was registered with PROSPERO.

### 2.2. Information Sources and Search Strategy

We investigate the PubMed and Scopus databases to identify studies published from September 2019 to May 2022. The generic free-text search terms were: “COVID-19”, “COVID”, “coronavirus”, “coronavirus infections”, “HCoV”, “2019 nCoV”, “nCoV”, “SARS”, “SARS-CoV-2”, “SARS coronavirus2”, “upper respiratory”, “pneumovirinae”, “pneumovirus infections”, “pneumonia”, “severe acute respiratory syndrome”, “co-infection”, “influenza”, “Superinfection”, “metapneumovirus”, “chlamydia”, “enterobacter”, “bacteria”, “fungus”, “respiratory syncytial viruses”, “respiratory virus”, “bacterial Infections”, “fungal infection”, “oxygen inhalation therapy”, and “viral infection”. We applied filters to include only full-text studies in English language, about human population, and to exclude reviews, systematic reviews, meta-analysis, autobiographies, historical article, interview and veterinary studies from the research. All records identified using our search strategy were exported to EndNote software.

For each study included in the review, the following information was collected in an Excell sheet: surname and first name of the first author, year of publication, pathogens identified, type of infection. RevMan 5.0 software was used for the meta-analysis.

## 3. Results

### 3.1. Study Selection

A total of 10 duplicates have been identified and removed from the 12.110 preliminary results. In total, 11.426 articles have been removed after looking at the title and abstract only since they did not match the criteria. A further 90 articles were excluded as the full text was not found. The remaining papers (*n* = 575) have been selected or excluded after a comprehensive analysis of the full text. Among those papers, 17 were excluded as these were reviews and 1 because it concerned an animal trial. The screening of the literature was performed in blind by two investigators (R.S. and E.B.) In the case of disagreement, a third reviewer (F.D.M.) assessed the paper to achieve a consensus.

**Figure 1:**
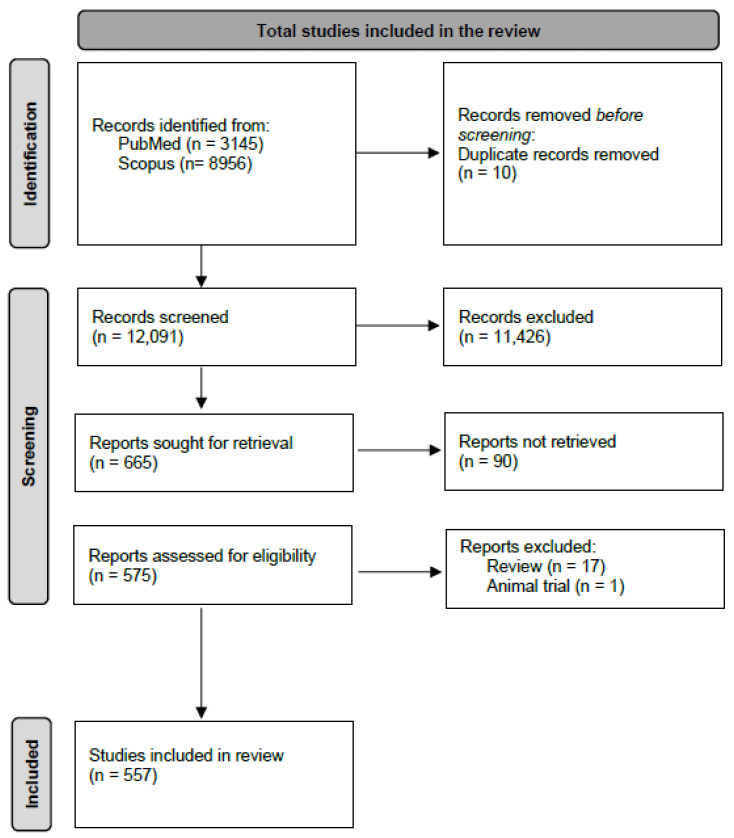
Flowchart showing the process of inclusion of publications.

Figure 1 summarizes the results of the study selection, represented as a PRISMA flow diagram obtained following the guidelines published by Page and colleagues [23].

Eight studies were selected for the meta-analysis because they showed specific information on the number of cases of co-infection or super-infection in SARS-CoV-2-infected and SARS-CoV-2-uninfected patients, together with the number of SARS-CoV-2-infected and SARS-CoV-2-uninfected patients without co-infection or super-infection [24,25,26,27,28,29,30,31].

### 3.2. Results of Syntheses

The articles considered were published from 2020 to 2022. In total, 121 studies were retrospective studies regarding co-infection and/or super-infection of more than one pathogen, 532 studies were about co-infection, 19 studies were about super-infection and 6 studies were about co-infection and super-infection. There was a total of 456 articles are case studies regarding the research of co-infection or/and super-infection of one specific pathogen. The data presented in this study are available in supplementary materials (Table S1).

**Figure 2:**
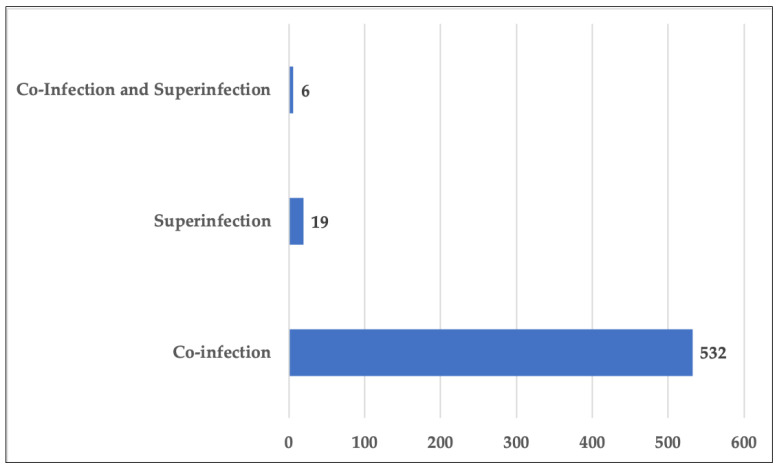
Number of co-infection, co-infection and super-infection, super-infection in the selected studies.

Of the selected studies, 532 studies showed that the infection occurred ≤48–72 h after hospital admission, 19 studies reported that the infection occurred >48–72 h after admission, and 6 studies illustrated a co-infection and a super-infection. There were also infections previous to hospitalization (Figure 2).

**Figure 3:**
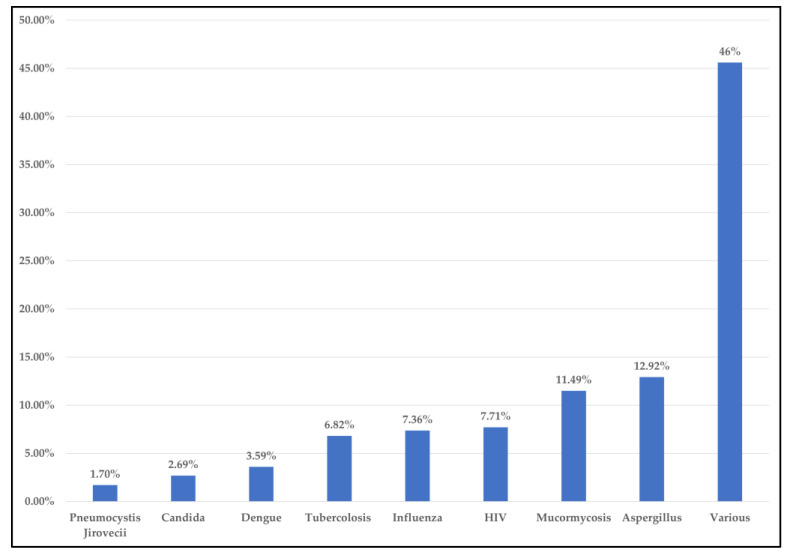
Most frequent pathogens in co-infection, super-infection and infections previous to hospitalization.

We clustered the studies according to the type of pathogen identified, placing a pathogen identified in less than 10 of the selected studies in ‘various’. The most commonly identified pathogens were Aspergillus (12.9%), Mucormycosis (11.49%), HIV (7.71%), Influenza viruses (7.36%), Tuberculosis (6.82%), Dengue (3.59%), Candida (2.69%), Pneumocystis Jirovecii (1.70%). In 46% of cases, the pathogens identified appeared in fewer than 10 studies (Figure 3).

Among the super-infections, the most frequent pathogens identified were Mucormycosis (31.50%), Aspergillus (21%), Tuberculosis (10.50%) and Staphylococcus Aureus (5.20%). In the remaining 31.5% of cases, various pathogens including bacteria, fungi and viruses were identified.

### 3.3. Meta-Analysis

For the meta-analysis, we grouped the cases according to Table 1, also considering the main findings and pathogens involved in patients with and without SARS-CoV-2 infection.

The purpose of the meta-analysis conducted is to verify whether positivity to COVID-19 is a risk factor for the development of co-infections/super-infections in hospitalized patients.

The studies included in the meta-analysis showed that respiratory pathogen infection rates were significantly higher in COVID-19 patients than in non-COVID-19 patients [24].

In addition, they highlighted some significant aspects concerning risk factors and the development of the disease. In particular, advanced age and being in nursing home care were associated with higher rates of co-infection [24]. Multivariable predictors of mortality were age, tachypnoea, hypoxaemia and bacterial infection. With regard to the latter, most infections in COVID-19 patients were nosocomial compared to community-acquired infections in non-COVID-19 [29]. COVID-19 co-infected patients were more likely to be male, were hospitalised more frequently, were more likely to die and were younger at the time of death [26].

In contrast, chronic diseases such as HBV infection did not predispose patients with COVID-19 to more severe outcomes [28] and patients with Candida auris co-infection did not have a dissimilar mortality rate compared to those without Candida auris [30].

**Figure 4:**
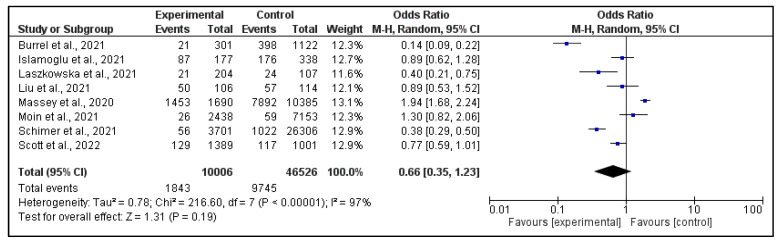
Forest plot for random effects meta-analysis about occurrence of infections associated with COVID-19 infection [24,25,26,27,28,29,30,31].

The results of the meta-analysis are reported in Figure 4. The results of our meta-analysis are expressed in terms of odds ratio. The overall result indicates that having a SARS-CoV-2 infection does not represent a real risk for the development of co-infections/super-infections; on the contrary, it would seem to paradoxically be almost a “protection” factor. Previous reports have documented a significantly higher risk of microbial infection in patients infected with SARS-CoV-2 infection. However, the currently published literature showed a substantial variability concerning the rate of co-infection and super-infection in patients with SARS-CoV-2 [32].

Meta-analyses attempted to identify the prevalence of bacterial co-infections among COVID-19 patients. In 2020, Langford et al. identified 24 retrospective studies; the bacterial co-infection rate was 3.5%. [32].

## 4. Discussion

Measures to contain the transmission of SARS-CoV-2 have also proven effective in reducing the transmission of other endemic respiratory viruses. The clinical outcome of respiratory viral co-infections with SARS-CoV-2 is unknown [33].

Bacterial co-infections make a formidable contribution to increased morbidity and mortality during seasonal influenza pandemics and epidemics. Bacterial co-infections cause an increase in hospital admissions, a greater severity of the clinical picture and an increase in mortality. The rate of co-infections increases in patients admitted to intensive care units. Gradual antibiotic therapy should be respected to avoid the emergence and spread of antibiotic-resistant bacterial strains. Co-infection with Mycobacterium tuberculosis and fungi is also described in a minority of patients infected with SARS-CoV-2 [34].

To our knowledge, our study is the first systematic review to evaluate the protagonist of co-infections or super-infections in patients with confirmed SARS-CoV-2 infection.

The results of the meta-analysis are not conclusive for the outcomes studied, although a tendency was found for co-infection in persons with COVID-19 by other pathogens such as S. aureus, S. pneumoniae, H. influenzae, M. catarrhalis, and K. pneumoniae [35].

Among the many organisms identified, special attention should be paid to S. aureus, S. pneumoniae, H. influenzae, M. catarrhalis, K. pneumoniae. These microorganisms are colonisers of the upper respiratory tract and can increase the risk of invasive infections and serious complications. In COVID-19 patients requiring intensive care, oropharyngeal colonisation could be a potential cause of ventilator-associated pneumonia (VAP), causing increased hospital and ICU stays. Therefore, it may be appropriate to perform further diagnostic tests to identify these pathogens and prevent a deterioration in the health of COVID-19 patients [36].

In COVID-19 patients admitted to intensive care units (ICUs), SARS-CoV-2 may facilitate the colonisation and attachment of bacteria to host respiratory tissue, leading to mixed infections. On the other hand, a bacterial super-infection may facilitate systemic spread of the virus, increasing the risk of septic shock [36,37]. A circular pathogenic mechanism of concausal type is realised, resulting in the deterioration of the COVID-19 patient’s health until eventual death from septic shock.

The aforementioned pathogenic mechanism plays an important role in the outcome of COVID-19 patients considering that up to 25 per cent of hospitalised and non-hospitalised COVID-19 patients may present with super-infections, with unfavourable therapeutic outcomes, including increased mortality. [38].

The rate of bacterial co-infection among COVID-19 patients ranges from 3.5% to 7–8% [32,39,40], although the data are the product of studies with limited patient samples where there was no clear definition of co-infection. More recently, Musuuza et al. retrieved the data of 118 studies that covered both ICU and non-ICU patients. The pooled analysis revealed that the prevalence of bacterial co-infection was 8% (95% CI: 5–11%); the rate of co-infections was higher amongst non-ICU patients [38]. The observed heterogeneity in the published literature concerning the rate of bacterial co-infections may be attributed to the variations in the definition of co-infection, study design, and the study periods.

The outcome for patients who contract a co-infection or super-infection is certainly pejorative; however, the question addressed in this study does not concern the prognosis but the risk of contracting hospital co-infections or super-infections. Could it be that the inflammatory mediators generated by the SARS-CoV-2 infection prepare the body to increase its defence against other pathogens?

On the one hand, the COVID-19 pandemic has significantly influenced the work of forensic pathologists [40,41]. On the other hand, forensic pathologists played an important role in the assessment of causes of death during the COVID-19 pandemic [42].

Determining whether a person died as a result of COVID-19 or whether other medical conditions contributed to his or her death made it possible to improve patient management and treatment strategies [43]. This can be important in helping to understand the effectiveness of disease prevention and control strategies, as well as in ensuring the correct attribution of causes of death [44]. COVID-19 patients may develop chronic pulmonary complications [45] or other diseases as a result of the infection [46,47,48,49]. Forensic pathology can contribute to the understanding of these complications and the development of appropriate therapies, and provide guidelines on how to manage deaths safely and effectively to protect both staff and the public [50].

Analysis of the presence of co-pathogens in the future will help the optimal diagnosis and treatment of suspected SARS-CoV-2 respiratory infections in the pandemic [24].

Our study shows that the most frequent super-infections of the respiratory system are aspergillosis, an infectious disease caused by inhalation of the opportunistic fungus Aspergillus, which it is characterised by a clinical picture ranging from asymptomatic to life-threatening [51], and Mucormycosis, a syndrome caused by mycetes of the genus Absidia, Mucor and Rhizopus, which occurs in the presence of favourable conditions, the most important of which is acidosis [52].

The studies involved in the meta-analysis showed the risk factors associated with the development of co-infection: male gender, advanced age, care in a nursing home [24,26]. In addition, age, tachypnoea, hypoxaemia and bacterial infection are predictors of mortality [31]. Nevertheless, the results of the meta-analysis show that having a SARS-CoV-2 infection does not represent a real risk for the development of co-infections/super-infections; on the contrary, it would paradoxically almost appear to be a ‘protective’ factor.

The strengths of our study include the use of a comprehensive and meticulous search strategy to identify potentially eligible studies from multiple databases and the manual search of recently published articles up to the search deadline.

However, there are methodological limitations that need to be considered. At present, there have been 758,390,564 confirmed cases of COVID-19 globally, including 6,859,093 deaths [53]. However, compared to the number of studies considered eligible in this review, this is only a small representation of the total number.

Evidence suggests that the overuse of broad-spectrum antibiotics may adversely affect the sensitivity of bacterial culture methods and the ability to detect co-infections [54]. Therefore, for the patients included in this review, the administration of broad-spectrum antibiotic therapy may have decreased the sensitivity of bacterial culture methods, resulting in an underestimation of the number of co-infections.

The presence of bacteria in microbiological cultures does not always indicate a genuine secondary infection; however, it could be the result of normal colonisation of our bodies or culture errors [55]. Some of the bacteria reported in the studies analysed may not be the causative agents of secondary infections but the colonisation of a normally non-sterile site. Furthermore, the presence of some bacteria in microbiological cultures may be the result of a contaminated sample or culture error, rather than an actual infection.

Furthermore, there is no comprehensive information on the pre-existing clinical condition of SARS-CoV-2 infection and how the presence of co-morbidities could be a predisposing factor to the development of co-infections or super-infections.

Each variant of COVID-19 could have a different impact on the patient, including the development of co-infections or super-infections [56,57]. The immune escape process of the Omicron variant could lead to prolonged viral shedding and increase hospitalization times in more compromised patients, with an increased risk of pulmonary co-infections or super-infections [58].

Further variables include antibiotic resistance [59] and the level of appropriateness of care and drug prescription also guaranteed by the availability of healthcare professionals such as clinical pharmacists [60]. The order of co-infection or super-infection should also be considered, as the level of the innate immune response elicited by the first pathogen may condition the second infection and the outcome of infections [61] must be considered.

## 5. Conclusions

Forensic pathology plays a crucial role in the management of the COVID-19 pandemic, providing important information on diagnosis, assessment of causes of death and management of deaths. This review shows a co-infection in 95% of the cases, a super-infection in 3.4% and a co-infection and a super-infection in 1%. However, even with the aforementioned limitations, it appears that SARS-CoV-2 infection does not represent a real risk for the development of co-infections/super-infections.

This systematic review is an accurate synthesis of the best available evidence; however, it is also a contribution to this field of study. Nevertheless, there are inherent research biases that need to be considered and require further investigation.

## Figures and Tables

**Table 1:** Characteristics of the studies used in the meta-analysis.

Author (Year)	IC	NIC	ICON	NICON
Massey BW et al. (2020) [24]	1453 in 1690	237 in 1690	7892 in 10,385	2493 in 10,385
İslamoğlu MS et al. (2021) [25]	87 in 177	90 in 177	176 in 338	162 in 338
Schirmer P et al. (2021) [26]	56 in 3701	3645 in 3701	1022 in 26,306	25,284 in 26,306
Burrel S et al. (2021) [27]	21 in 301	280 in 301	398 in 1122	724 in 1122
Liu R et al. (2021) [28]	50 in 106	56 in 106	57 in 114	57 in 114
Moin S et al. (2021) [29]	26 in 2438	2412 in 2438	59 in 7153	7094 in 7153
Laszkowska M et al. (2021) [30]	21 in 204	183 in 204	24 in 107	83 in 107
Scott H et al. (2022) [31]	129 in 1389	1216 in 1389	117 in 1001	884 in 1001

IC: Number of co-infection cases in SARS-CoV-2 infected; NIC: Number of not co-infection cases in SARS-CoV-2-infected; ICON: Number of infection cases in not-SARS-CoV-2-infected; NICON: Number of not-infection cases in not-SARS-CoV-2-infected.

## Data Availability

Not applicable.

## Supplementary Materials

The following supporting information can be downloaded at: https://www.mdpi.com/article/10.3390/pathogens12050646/s1, Table S1: Characteristics of the studies included in the review.
